# Community contexts and knowledge: accountability, leadership and meaningful involvement of communities in Programme Science

**DOI:** 10.1002/jia2.26281

**Published:** 2024-07-10

**Authors:** Ruth Morgan Thomas, Andrew Spieldenner, Erika Castellanos, Judy Chang, Jules Kim

**Affiliations:** ^1^ Independent Consultant Edinburgh UK; ^2^ MPact Global Action for gay men's health and rights San Francisco California USA; ^3^ Global Action for trans equality New York City New York USA; ^4^ International Network of People who Use Drugs London UK; ^5^ Global Network of Sex Work Projects Edinburgh UK

**Keywords:** community, health systems, HIV care continuum, human rights, key and vulnerable populations, structural interventions

## Abstract

**Introduction:**

A Programme Science approach that prioritizes populations who will benefit most and ensuring resources are allocated to programmes that meet the needs of those populations will bring an equity focus to research. Gay men and other men who have sex with men, people who use drugs, sex workers of all genders, and trans and gender‐diverse people, defined by the Joint United Nations Programme on HIV/AIDS (UNAIDS) and The Global Fund to Fight AIDS, Tuberculosis and Malaria (Global Fund) as key populations, have been disproportionately impacted since the start of the HIV pandemic. Through documenting community experiences from global key population‐led networks, the authors explore the potential value and impact of community‐led organizations and service delivery as critical components in effective HIV and Sexually Transmitted infections (STI) programmes.

**Discussion:**

Through advocacy and research interventions, global key population networks have identified barriers against scaling up interventions for criminalized and marginalized communities, as well as highlighted solutions. The authors examine some of the current barriers to meaningful involvement of communities and the scaling up of community‐led programmes that need to be addressed if Programme Science is to maintain an equity lens and the needs of key populations are to be met and highlight the need to make visible community engagement and participation in embedded research and Programme Science.

**Conclusions:**

The Programme Science approach provides an important opportunity to understand practical issues that will increase effective coverage in the implementation of public health and other interventions, which will require the prioritizing of key populations and their priorities in HIV and STI programmes. It will require extensive time and work to build relationships, increase capacity and share power. Where this has already happened, it has resulted in positive outcomes, including better health outcomes, reduced stigma, increased agency for key populations, and built community‐led organizations and responses.

## 1 INTRODUCTION

A Programme Science approach that prioritizes populations who are most vulnerable to poor health outcomes will bring an equity focus to Programme Science and research and ensure resources are allocated to programmes that meet the needs of those populations thereby achieving the greatest benefit. If health inequities are to be addressed, and efficacy improved, then we need to understand which communities are at increased vulnerability of poor sexual and other health outcomes, and what drives the disproportionate vulnerability of some populations. Programme Science requires community engagement as the means to develop, implement and distribute knowledge. Through understanding community concerns, Programme Science often addresses larger issues beyond behaviour change or biomedical interventions. This paper focuses on community‐led organizations as key partners in Programme Science.

Key populations are defined in HIV by UNAIDS and the Global Fund to be: gay men and other men who have sex with men, people who use drugs, sex workers of all genders, and trans and gender‐diverse people. Key population communities around the world recognized their vulnerability early in the HIV pandemic and began organizing to advocate for an equity and human rights‐based approach to HIV and STI programming. Decades of community mobilization has led to strong grassroots community‐led groups and organizations, and the development of national, regional and global key population‐led networks. Even still, HIV continues to have a disproportionate impact on key populations.

Significant progress has been made in reducing the number of new HIV acquisitions across the world, including preventing new HIV acquisitions among some groups, such as adolescent girls and young women. Adolescent girls and young women are considered a separate and distinct population for programming even though key population communities include many adolescent girls and young women. These HIV prevention successes have not had the same impact among key populations as the general public, and HIV disparities persist. While the four key populations account for less than 5% of the global population, they were reported to account for 46% of new acquisitions in 2018, 54% in 2019 and 65% in 2020, with the latest UNAIDS Global AIDS Update reporting that 70% of new HIV acquisitions in 2021 were among key populations and their sexual partners [1].

Underpinning this failure to serve key populations equitably is stigma and discrimination in both healthcare and law enforcement settings. This is exacerbated and sustained by the direct and indirect criminalization of key populations. As a consequence, key population communities are too often excluded from meaningful involvement in discussions and decision‐making about policies and programmes that impact their health and wellbeing. Addressing community priorities, such as stigma and discrimination, could significantly reduce poor health outcomes yet these priorities are not considered critical components of HIV and STI programmes. It is, therefore, essential that public health authorities, policymakers and implementers look beyond established knowledge canons, such as randomized control trials (RCTs), to understand the importance of meaningful involvement of communities in the assessment, development, implementation and evaluation of interventions.

The Programme Science approach can bring about a paradigm shift and explicitly centre key population communities recognizing and utilizing their expertise and knowledge through meaningful involvement in the development of Prevention Strategies. The other articles in this supplement on Programme Science explore how centring communities in the learning and research agendas and methodologies ensure HIV programmes are accessible, effective and considered acceptable by the targeted key populations. In this commentary on Programme Science, we highlight some challenges that key population communities have faced in global health, and some barriers Programme Science can face centring key population communities.

## 2 DISCUSSION

Key population‐led organizations and networks are founded on and driven by priorities identified by their communities, which include the health, safety and wellbeing of individuals, as well as building the community's capacity to develop and deliver services that meet their needs and engage with policymakers and implementers. In too many contexts, policymakers and implementers focus primarily on changing behaviour that they consider “undesirable” without fully understanding the implications and consequences for either individuals or the community.

The COVID‐19 pandemic presented a moment in time when across the world everyone felt vulnerable, where the opportunity to prioritize saving lives was presented and policy challenges could be addressed. It was during the pandemic that key population‐led organizations demonstrated again their ability to respond to community needs when many governments failed to include key populations in emergency responses and national social protection mechanisms. Some countries even used the COVID‐19 pandemic to aggressively target key populations with extraordinary legal oppression in the name of public health, including: closing down sex work businesses; demolishing buildings which were also sex workers’ homes leaving them and their families without any means of financial support and homeless [2]; increased surveillance and harassment of people who use drugs by law enforcement, accompanied by increased violence, particularly against women who use drugs who continued to be denied access to shelters [3]; and mobility restrictions were introduced based on gender markers on identification documents, which did not permit mobility for transgender, gender non‐conforming and intersex people, denying them the possibility to safely leave their homes to buy food or collect medication, and making them even more vulnerable to state violence at police checkpoints [4]. While governments strived to maintain HIV treatment access, they failed to respond to other needs key populations faced in the pandemic.

In response to the lack of support from governments, key population communities and key population‐led organizations advocated with local and national policymakers and programmers, as well as internationally with UN bodies to demand that key populations not be left behind in the COVID‐19 response. Given the intentional exclusion of key populations from most national responses, community‐led organizations successfully developed their own emergency responses, including the distribution of food parcels and personal hygiene supplies, the establishment of mutual aid funds, the development and implementation of innovative ways to ensure uninterrupted access to HIV prevention technologies and treatment, such as extended prescribing to avoid frequent trips to clinics and pharmacies (and thus risk COVID‐19 exposure). People who use drugs and their networks were critical in the response to mitigate the impact of COVID‐19 on their communities by publishing harm reduction guidance in the context of COVID‐19, designed innovative overdose prevention initiatives, successfully advocated for policymakers and programmes to initiate take‐home doses of opiate agonist treatment (in some contexts for the first time), educated shelters and quarantine centres on the needs of people who use drugs and linked them up to relevant resources, and pushed law enforcement to halt arrests and harassment of street‐based populations [5]. These examples from COVID‐19 demonstrate how challenges key population communities faced in HIV remained during another health crisis. The structural barriers highlighted here must be central to health research, policy and services.

The over‐reliance on scientific and medical research methods such as RCTs does not allow for documenting the complexity of context and experiences that impact upon key population's vulnerability to HIV. In order to be effective as a model of research, policy and service development, Programme Science needs to do better and more about including structural interventions particularly those addressing stigma and discrimination and the impact of criminalization. The few research papers on peer involvement and leadership that have been published have documented positive results and impacts. A scoping review of 279 papers found more than 40 beneficial outcomes linked to a range of peer and community‐led HIV activities:—including improved HIV‐related knowledge, attitudes, intentions, self‐efficacy, risk behaviours, risk appraisals, health literacy, adherence and viral suppression; improvements in HIV service access, quality, linkage, utilization and retention resulting from peer‐ or community‐led programmes or initiatives; structural level changes, including positive influences on clinic wait times, treatment stockouts, service coverage and exclusionary practices [6]. These studies demonstrate the comparative advantage of peer‐ and community‐led HIV responses and are further supported by the targets on community‐led responses set out in the Global AIDS Strategy and 2021 Political Declaration on HIV/AIDS. Ensuring community‐led responses and programmes are integrated into the Programme Science approach, and their contribution is documented, will provide further evidence of the impacts and outcomes and support the scaling up of key population‐led programmes which will address current inequities.

Programme Science must meaningfully involve key population‐led organizations by ensuring that they are involved from the design of the research agenda through to the analysis of data and the reviewing and endorsing of the conclusions drawn prior to publication. Programme Science, therefore, requires the requisite time and other resources (including materials translation, development of collaterals, paying for community members’ time and expertise utilized in processes) that would result in meaningful engagement. Further, key population‐led organizations have the right to determine who represents them, and should have the ability to affect the flow of research (e.g. identifying harms, collaborating on solutions and developing interventions) [7].

### 2.1 Barriers to scaling up community‐led programmes

Key population‐led organizations have demonstrated their ability and commitment to serve their communities and achieve impressive results, but rarely have they been resourced to scale up programmes or their experiences integrated into research and learning agendas for HIV and STI prevention and care programmes. Regretfully, most donors, implementers and public health authorities rely on RCTs to establish the impact of interventions in their HIV and STI programmes; and community impact is not considered as valid without RCTs. These investments exclude communities, as RCTs require multiple levels of institutional capacity and support. Those same levels can present barriers in including key populations, who may not be comfortable in the site, willing to engage in the RCT restrictions or may be endangered when put in the control arm of the RCT. The RCT as the “gold standard” of research can thus present a tautology of exclusion for many key populations. While communities have monitored and documented the positive impacts of their interventions, they rarely if ever use RCTs, particularly for interventions that seek to address human rights; the Programme Science approach can ensure such evidence is included and valued.

Multi‐ and bi‐lateral donors’ investment in programmes to address human rights barriers that exacerbate key population communities vulnerability to HIV and STIs is too often given to governments, a strategy that has been supported by UN agencies which justify it by arguing that governments bear the responsibility as duty bearers to protect all human rights. However, too often those same governments criminalize key populations, which emboldens perpetrators of human rights violations and allows them to act with impunity. One such example is The Global Fund Breaking Down Barriers Initiative started in 2017 in order to address structural barriers that limit the reach and impact of key and vulnerable population programmes. Funds went to governments to specifically address structural barriers for key populations, often millions of USD. While there has been traction in some countries, Indonesia, Uganda, Ghana and Kenya have all put forward anti‐LGBTQ legislation in this time frame, with more countries following [8]. Key population communities, who have been criminalized and faced extreme stigma and discrimination for centuries in many contexts, have out of necessity developed strategies to minimize the impact of such structural barriers and have the expertise and knowledge to break down the barriers that exclude them from better health outcomes.

### 2.2 Barriers to be overcome for meaningful involvement of communities in Programme Science

Stigma and discrimination impact people's ability and willingness to engage in healthcare and access health and social care services. This can lead to poor health outcomes, including detectable HIV viral load, lapse in prevention and harm reduction coverage, and other STIs. Evidence is accumulating that stigma and discrimination contribute to the gap in effective coverage, Programme Science must directly address and seek to mitigate stigma and discrimination through gathering and using data to inform programme design and implementation.

If Programme Science is to achieve the goal of high‐impact embedded research initiatives, then addressing stigma and discrimination and the impact of criminalization must be integrated and key population community members must be seen as more than data, subjects of research or data gatherers. It is important that key population‐led organizations and communities are seen as equal partners with meaningful involvement at every step of the process. Implementing Comprehensive HIV/STI Programmes with Sex Workers, also known as the Sex Worker Implementation Tool published by UNAIDS, Co‐sponsors and Global Network of Sex Work Projects highlights the importance of addressing barriers to the meaningful involvement of key populations “The meaningful participation of sex workers is essential to building trust and establishing relationships and partnerships that have integrity and are sustainable. This may be challenging for service providers [and the UN, INGOs, NGOs, Bilateral Programmes, Governments, and the Global Fund] who are more accustomed to establishing the parameters within which services are provided, and prescribing how relationships or partnerships are to be conducted. As sex workers and sex worker organizations become more empowered, there will be greater expectations of power‐sharing and power‐shifting [9].” Transparent processes for decision‐making include time for community consultation with comprehensive information made available in a timely manner and in the languages spoken by the key population communities in the country (including migrants). Key population‐led organizations and communities must be able to choose how they are represented, and by whom; whether to participate or not; how they engage in the process; and have equal voice in how the partnerships are managed. Through meaningful involvement of key populations, we can (and must) address issues of stigma and discrimination.

### 2.3 The importance of shared knowledge production

Communities should have ownership in the process of knowledge production. The meaningful involvement of key population‐led organizations and communities in the design, implementation and analysis of embedded research is critical. Programme Science's engagement premise must be made visible and communities must be able to use the data they gathered in community advocacy. Key populations are often very experienced with being research subjects, but rarely participate beyond that. Researchers do not often check and discuss findings before publication with the communities they study. This might include translating findings without jargon for community members to respond, challenge and possibly make demands.

Key population‐led organizations can also extend the reach and impact of the research findings, as they have extensive experience in making research findings accessible to their communities across communication platforms such as social media, drop‐in centres, websites and through investment in the creation of multiple media such as 30‐ and 60‐second videos, art advocacy and cartoons to transmit key findings.

## 3 CONCLUSIONS

The Programme Science approach provides an important opportunity to ensure meaningful involvement of community‐led organizations that will allow for greater understanding and increase effective coverage in the implementation of public health and other interventions, as demonstrated during the COVID‐19 pandemic. It will require the prioritizing of key populations and addressing their priorities in HIV and STI programmes. It will also require extensive time and work to build relationships, increase capacity and share power. Where this has already happened, it has resulted in positive outcomes, such as better health outcomes (including treatment adherence), reduced stigma, increased agency for key populations, and built community‐led organizations and responses [10].

We recommend:

- Increasing understanding of cultures, community, contexts and challenges of key populations;
- Engaging key population‐led organizations rather than individuals in order to ensure that the needs of the communities are centred;
- Respecting community knowledge about the issues that impact them, even if they express their concerns and questions in different ways; and
- Investing in long‐term partnerships with key population‐led organizations in every stage of programme design, research, implementation, management and evaluation.

## COMPETING INTERESTS

None of the authors have any competing interests.

## AUTHORS’ CONTRIBUTIONS

RMT, AS, EC, JC and JK conceptualized the paper. RMT wrote the first draft of the paper, AS and JC contributed in writing different sections of the paper and AS, EC, JC and JK reviewed drafts.

## Data Availability

Data sharing is not applicable to this article as no datasets were generated or analysed during the current study.
